# Life course plasma metabolomic signatures of genetic liability to Alzheimer’s disease

**DOI:** 10.1038/s41598-024-54569-w

**Published:** 2024-02-16

**Authors:** Hannah Compton, Madeleine L. Smith, Caroline Bull, Roxanna Korologou-Linden, Yoav Ben-Shlomo, Joshua A. Bell, Dylan M. Williams, Emma L. Anderson

**Affiliations:** 1 Bristol Medical School, Population Health Sciences, University of Bristol, Bristol, UK; 2 Medical Research Council Integrative Epidemiology Unit, University of Bristol, Bristol, UK; 3 School of Translational Health Sciences, University of Bristol, Bristol, UK; 4 MRC Unit for Lifelong Health & Ageing at UCL, University College London, London, UK; 5 Division of Psychiatry, University College London, 149 Tottenham Court Road, London, W1T 7NF UK

**Keywords:** Alzheimer’s disease, APOE, Polygenic risk score, Mendelian randomization, Metabolism, NMR, Epidemiology, ALSPAC, UK Biobank, Neurological disorders, Genomics

## Abstract

Mechanisms through which most known Alzheimer’s disease (AD) loci operate to increase AD risk remain unclear. Although Apolipoprotein E (APOE) is known to regulate lipid homeostasis, the effects of broader AD genetic liability on non-lipid metabolites remain unknown, and the earliest ages at which metabolic perturbations occur and how these change over time are yet to be elucidated. We examined the effects of AD genetic liability on the plasma metabolome across the life course. Using a reverse Mendelian randomization framework in two population-based cohorts [Avon Longitudinal Study of Parents and Children (ALSPAC, n = 5648) and UK Biobank (n ≤ 118,466)], we estimated the effects of genetic liability to AD on 229 plasma metabolites, at seven different life stages, spanning 8 to 73 years. We also compared the specific effects of *APOE ε4* and *APOE ε2* carriage on metabolites. In ALSPAC, AD genetic liability demonstrated the strongest positive associations with cholesterol-related traits, with similar magnitudes of association observed across all age groups including in childhood. In UK Biobank, the effect of AD liability on several lipid traits decreased with age. Fatty acid metabolites demonstrated positive associations with AD liability in both cohorts, though with smaller magnitudes than lipid traits. Sensitivity analyses indicated that observed effects are largely driven by the strongest AD instrument, *APOE*, with many contrasting effects observed on lipids and fatty acids for both ε4 and ε2 carriage. Our findings indicate pronounced effects of the *ε*4 and ε2 genetic variants on both pro- and anti-atherogenic lipid traits and sphingomyelins, which begin in childhood and either persist into later life or appear to change dynamically.

## Introduction

By virtue of our ageing population, the number of patients with Alzheimer’s disease (AD) continues to rise^1^. Neuropathological hallmarks of AD precede the onset of clinical symptoms by decades^2^, yet diagnosis is often late in the disease course. Brain and cerebrospinal fluid (CSF) biomarkers discriminate AD cases from controls with high accuracy^3,4^, though sample collection is invasive. Thus, great impetus remains for identification of more easily measured plasma AD biomarkers, which could improve our understanding of early disease aetiology.

AD involves a complex genetic architecture. Genome-wide association studies (GWAS) have illuminated many AD-associated single nucleotide polymorphisms (SNPs); the largest to date identifying independent 75 risk loci^5^. The apolipoprotein E (APOE) ε4 allele (UK allele frequency 0.15), encoding an isoform of Apolipoprotein E (ApoE), greatly elevates AD risk, accounting for ~ 50% of total genetic susceptibility^6^. The APOE ε2 allele (UK allele frequency 0.8) reduces AD risk by up to 87% compared to ε3 homozygotes^7^. Given that ApoE functions to regulate lipid homeostasis^8^, it is postulated that circulating lipid perturbations are associated with both AD risk and early pathology. Indeed, lipidomic studies suggest that both increased and decreased cholesterol, phospholipids, and sphingolipids^9^ may reflect neurodegeneration-associated membrane changes^10^. Many studies are, however, underpowered and given evaluation of AD patients in case–control studies, we cannot ascertain whether metabolic derangements are a cause or a secondary consequence of disease (i.e. biased by reverse causation), or confounded by lifestyle factors, medications or comorbidities such as cardiovascular disease (CVD)^10^. Considering other metabolic markers, glucose dysregulation is likely implicated in, or a reflection of, AD pathogenesis, given that abnormally low rates of glucose metabolism in *APOE4* carriers are observed decades before disease onset^11^. Serum amino acid profiles also accurately discriminate AD cases from controls^12^, hence impaired amino acid metabolism may contribute to AD pathogenesis or vice-versa^13^.

Using a reverse Mendelian Randomisation (MR) approach, this study aimed to characterise the metabolic features (both lipid and non-lipid) of higher genetic liability to AD, revealing early biomarkers of AD pathogenesis, which may be potentially targeted to prevent the clinical onset of AD. We constructed a genetic instrument for AD liability and examined the effects on circulating metabolites across the life-course, in two large population-based cohorts; the Avon Longitudinal Study of Parents and Children (ALSPAC) and UK Biobank. Finally, we performed a secondary analysis to further evaluate the potential molecular mechanisms underpinning the AD risk-increasing effect of *APOE* ε4 carriage, versus the protective effect of ε2 carriage, when compared with ε3 homozygosity.

## Methods

### Study participants

We used data from two UK population-based cohort studies. First, ALSPAC; a population-based multi-generational birth cohort study of 14,541 women and their offspring, from the Southwest of England^14,15^. Full details of ALSPAC are provided in the online supplement. ALSPAC offspring with the following information recorded were eligible for this study: genotype, sex, age, and at least one metabolic trait at any time point. A total of 5,648 individuals were eligible for analysis on at least one occasion. See Supplementary Fig. 1 for full details of the eligibility criteria and Supplementary Tables 1 and 2 for descriptive statistics of the eligible ALSPAC cohort. Second, we also used a combination of pre-existing summary-level GWAS data and novel analyses of individual-level data from UK Biobank; a large-scale multicentre cohort study of half a million UK participants aged 39–73 years at baseline assessments in 2006–2010. A total of 118,466 UK Biobank participants were included in these analyses. Full details of the UK Biobank design, participants, quality control and its strengths and limitations have been described previously^16–18^.

### Assessment of genetic liability to Alzheimer’s disease

In ALSPAC, genotypes were assessed using the Illumina HumanHap550 quad chip, with imputation performed with the Haplotype Reference Consortium panel^19^. AD liability was defined using weighted genetic risk scores (GRS) based on 25 SNPs associated with AD risk at genome-wide significance (p ≤ 5 × 10^−8^) reported by Kunkle et al.^20^ (n = 21,982 clinically diagnosed cases and n = 41,944 cognitively normal controls). This AD GWAS was chosen because it is the largest GWAS comprising only clinically diagnosed AD cases, not ‘by-proxy’ cases, which have been shown to cause bias in downstream analyses using GWAS summary data^21^. Risk-increasing alleles and log odds ratios from the final stage meta-analysis (Supplementary Table 3) were used as external weights. Data were harmonized such that the effect (risk-increasing) alleles were coded in the same direction in both the AD GWAS and ALSPAC data. One AD SNP (rs9331896, *CLU* gene) was not present in the ALSPAC dataset, thus, a proxy SNP in high linkage disequilibrium (LD) (within 10,000kb, r^2^ = 0.8) was used. AD-associated SNPs were combined into two GRSs; one including and one excluding the SNPs denoting the *APOE* isoforms to examine non-*APOE* driven effects. Given the missingness of genotype data for some ALSPAC participants, GRSs were created for all individuals with genotype data for at least one AD SNP to preserve sample size and statistical power. Over 90% of participants had 22 of the 25 AD SNPs, and the smallest number of SNPs for any included individual was 18 out of 25. In UK Biobank, genetic liability to AD was instrumented using the same SNPs used to create the GRSs in ALSPAC (i.e. including the same proxy SNP for CLU). As such, the same data harmonisation process was used.

### Assessment of metabolites

In ALSPAC, blood samples were taken at clinics when participants were approximately 8, 16, 18 and 25 years old. Samples were fasted except for those obtained at age 8 years. A total of 229 metabolites from a targeted metabolomics platform were measured via proton nuclear magnetic resonance (^1^H-NMR) spectroscopy using EDTA-plasma^22^. All metabolites were quantified at the first three time points; however, the following were not measured at 25 years: diacylglycerol, ratio of diacylglycerol to triglycerides, fatty acid chain length, degree of unsaturation, conjugated linoleic acid, and ratio of conjugated linoleic acid to total fatty acids. Most metabolites relate to lipoproteins, categorised by density and size. Lipoprotein characteristics are recorded, including their triglyceride, phospholipid and cholesterol content. Various fatty acid, glycolysis-related, amino acid and inflammatory trait concentrations are also included. In UK Biobank, non-fasting EDTA plasma samples from a random subset of participants (n = 118,466, phase one NMR release) were analysed for levels of 249 metabolites and ratios, using the same ^1^H-NMR platform as in ALSPAC, but with several additional ratios of lipid measures.

### Statistical approach

#### Primary analysis: effects of the AD GRS of the life course plasma metabolome

**Figure 1:**
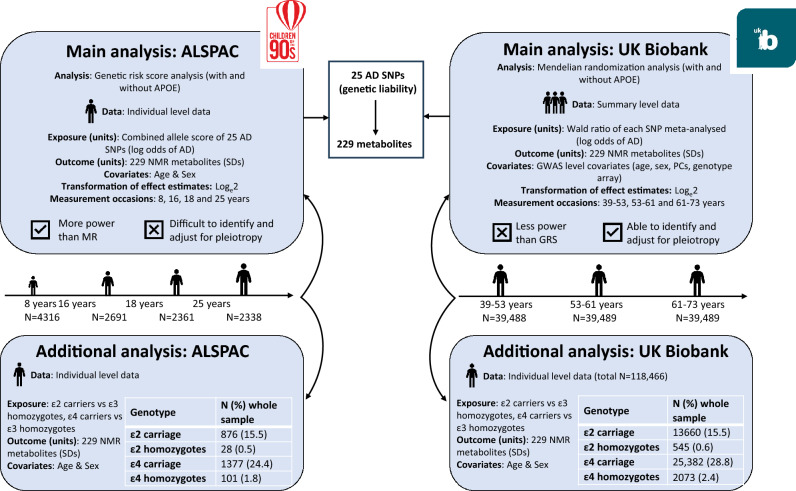
Illustration of the analytical models performed.

We adopted a reverse MR^23^ framework, such that genetic liability to AD is treated as the exposure and metabolites as the outcome, to ascertain the metabolic features of AD liability in a preclinical population. The reverse MR framework is useful for excluding reverse causation and confounding as potential explanations for any findings, because AD genetic variants are randomized at conception and, thus, cannot be altered by subsequent disease (both clinical and prodromal) and should not be confounded by lifestyle, social and behavioural factors. Figure 1 outlines the analytical methods performed.

In ALSPAC, we conducted a GRS analysis which combines alleles into a score. In the UK Biobank, we performed a formal MR analysis which uses SNPs as instrumental variables (IVs) for AD liability^24^ (i.e. generating a Wald ratio for each SNP and then meta-analysing them). GRS analyses are typically better powered than MR analyses and hence were more suitable for ALSPAC’s smaller sample size. GRSs do not, however, allow interrogation of potential bias due to horizontal pleiotropy. Though MR analyses are less well powered than GRS analyses, several sensitivity analyses (including MR-Egger, weighted median and weighted mode) enable the assessment of, and control for, horizontal pleiotropy^25^. To examine potential horizontal pleiotropy in the ALSPAC GRS analysis, we examined whether the AD GRS was associated with BMI, height, smoking, alcohol consumption, physical activity, maternal and paternal educational attainment, or maternal or paternal occupational social class (Supplementary Table 4).

Results across the two cohorts are directly comparable despite the different analysis methods; firstly, the exposure (AD liability) is on the log odds scale in both the GRS and the reverse MR (i.e. per log unit increase in AD liability). Secondly, the same standardizing transformation was applied to all metabolites. Thirdly, all effect estimates (from both GRS and MR) were multiplied by 0.693 (log_e_2), as recommended by Burgess et al.^26^ for binary/liability exposures, and estimates are therefore interpreted as SD-unit differences in each metabolic trait, per doubling of genetic liability to AD.

In both UK Biobank and ALSPAC analyses, all metabolites were standardized and normalized prior to analyses using rank-based inverse normal transformation. For the ALSPAC GRS analysis (n = 4316 at 8 years, n = 2691 at 16 years, n = 2361 at 18 years and n = 2338 at 25 years), associations between the AD GRS and each metabolite at each time point were assessed using separate linear regression models, adjusting for age at time of metabolite assessment and sex. Only eighteen percent (N = 779) of participants included in the age 8 analyses additionally had metabolites measured at the three subsequent time points. These analyses were performed in Stata Version 16.

For the main UK Biobank MR analysis, 118,466 participants of European ancestry were stratified into tertiles of age (youngest: 39–53 years. N = 39,488, middle: 53–61 years, n = 39,489, oldest 61–73 years, n = 39,489), before a GWAS of each metabolite was performed. Genetic association data for metabolites were generated using the MRC IEU UK Biobank GWAS pipeline^27^. SNP-exposure associations based on the same 25 SNPs for AD that were used to create GRSs in ALSPAC (i.e. including the proxy SNP for the CLU gene) were integrated with the SNP-metabolite associations. The following statistical methods were used to generate MR effect estimates using the TwoSampleMR package in R version 4.0.2^28^: inverse variance weighted (IVW), MR Egger, weighted median, and weighted mode, each making different assumptions about directional pleiotropy^29,30^. MR analyses were also repeated with a set of 23 SNPs excluding the two major *APOE* SNPs (rs7412 and rs429358), to compare with the ALSPAC GRS analysis.

### Additional analysis: comparing metabolic profiles of *APOE4* and *APOE2* with *APOE3*

As previous work in this area has shown that most associations observed between the AD GRS and downstream phenotypes are primarily driven by variation in the *APOE* locus, we conducted a further analysis to examine the molecular mechanisms that may underpin the risk-increasing effect of *APOE* ε4 carriage, and the protective effect of *APOE* ε2, when compared with *APOE* ε3 homozygosity. To do this, we used individual-level data from both ALSPAC and the UK Biobank. First, metabolic profiles in participants with at least one *APOE* ε4 allele (i.e. ε4 carriers) were compared to profiles in those who were *APOE* ε3 homozygous, omitting participants carrying an ε2 allele. Second, metabolic profiles in participants with at least one *APOE* ε2 allele (i.e. ε2 carriers) were compared to profiles in those who were *APOE* ε3 homozygous, omitting participants carrying an *APOE* ε4 allele. These models were based on multivariable linear regression, with binary independent variables for ε4 or ε2 carriage. Models were adjusted for age at follow-up and sex in ALSPAC. In keeping with the linear regression implementation of the GWAS pipeline used for the main analyses, the UK Biobank *APOE*—metabolite analyses were restricted to: (i) participants of European ancestry (as defined by the largest cluster following a K means clustering analysis of the top four genetic principal components); (ii) individuals with genotypic data passing quality control steps (no sex mismatches, aneuploidy, excess heterozygosity); (iii) individuals with no degree of kinship with other cohort members—one individual within pairs of the kinship matrix provided by the UK Biobank study team were randomly dropped. Models in UK Biobank included adjustments for age (within tertile), sex, genotype array and the first 10 genetic principal components provided by UK Biobank. This left a UK Biobank sample of n = 88,287, and an ALSPAC sample of n = 5648, prior to splitting into age tertiles and the omission of ε2 carriers from ε4 modelling and vice-versa. Sample sizes for the ε4 analysis in each age group were as follows: 8 years: N = 3452, 16 years: N = 2159, 18 years: N = 1871, 25 years: N = 1853, 39–53 years: N = 25,614, 53–61 years: N = 27,666 and 61–73 years: N = 21,347. For the ε2 analysis: 8 years: N = 3106, 16 years: N = 1928, 18 years: N = 1689, 25 years: N = 1695, 39–53 years: N = 21,420, 53–61 years: N = 23,363 and 61–73 years: N = 18,122. Supplementary Table 5 shows the number of heterozygotes and homozygotes for ε2 and ε4. Results are interpreted as the mean difference in metabolites in ε4 carriers and ε2 carriers, compared to ε3 homozygotes.

### Ethics approval

Ethical approval for the study was obtained from the ALSPAC Ethics and Law Committee and the Local Research Ethics Committees. Informed consent for biological samples has been collected in accordance with the Human Tissue Act (2004). UK Biobank has approval from the North West Multi-centre Research Ethics Committee (MREC) as a Research Tissue Bank (RTB) approval.

## Results

### Primary analysis: effects of AD liability on the life course plasma metabolome

**Figure 2:**
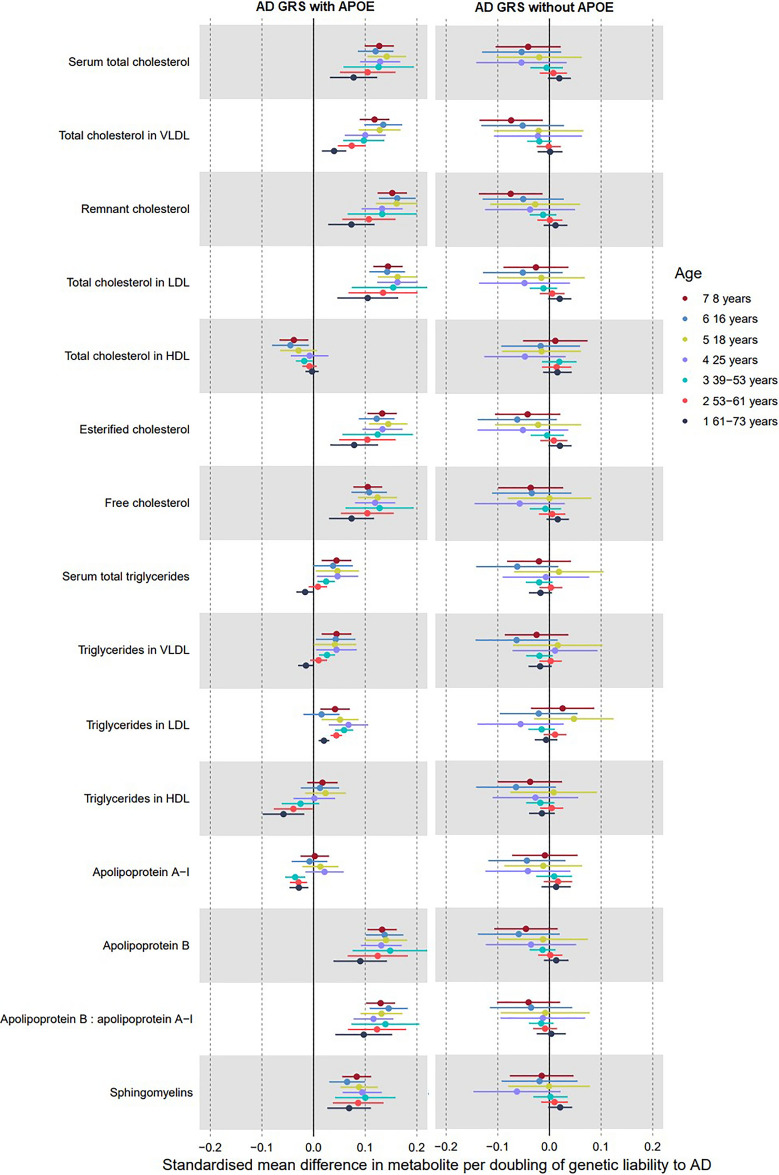
Forest plot showing the estimated effect of higher AD liability on main lipid metabolites (left panel including *APOE* variants; right panel excluding them). UK Biobank MR estimates are from the inverse variance weighted model.

**Figure 3:**
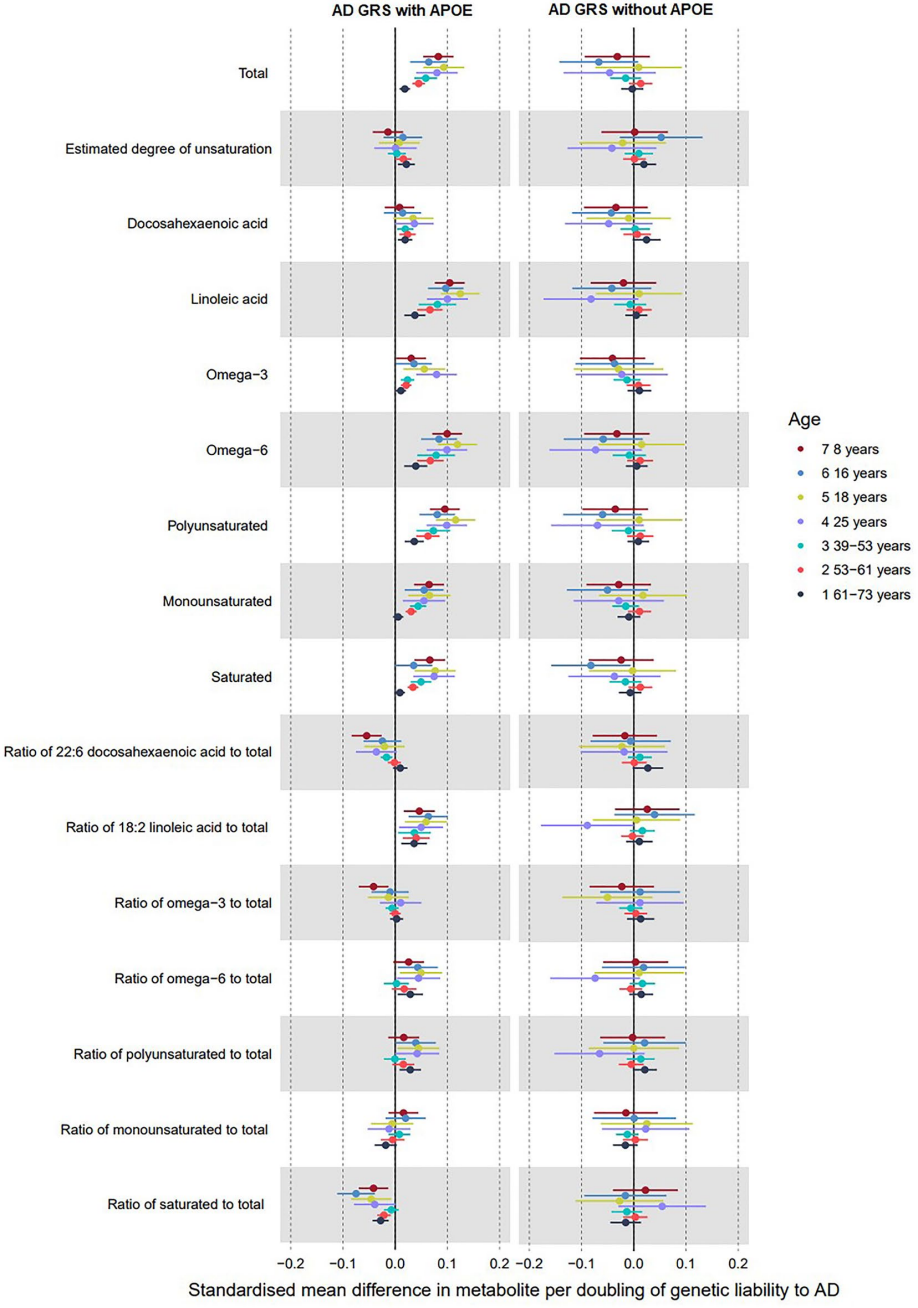
Forest plot showing the estimated effect of higher AD liability on main fatty acid metabolites (left panel including *APOE* variants; right panel excluding them). UK Biobank MR estimates are from the inverse variance weighted model.

**Figure 4:**
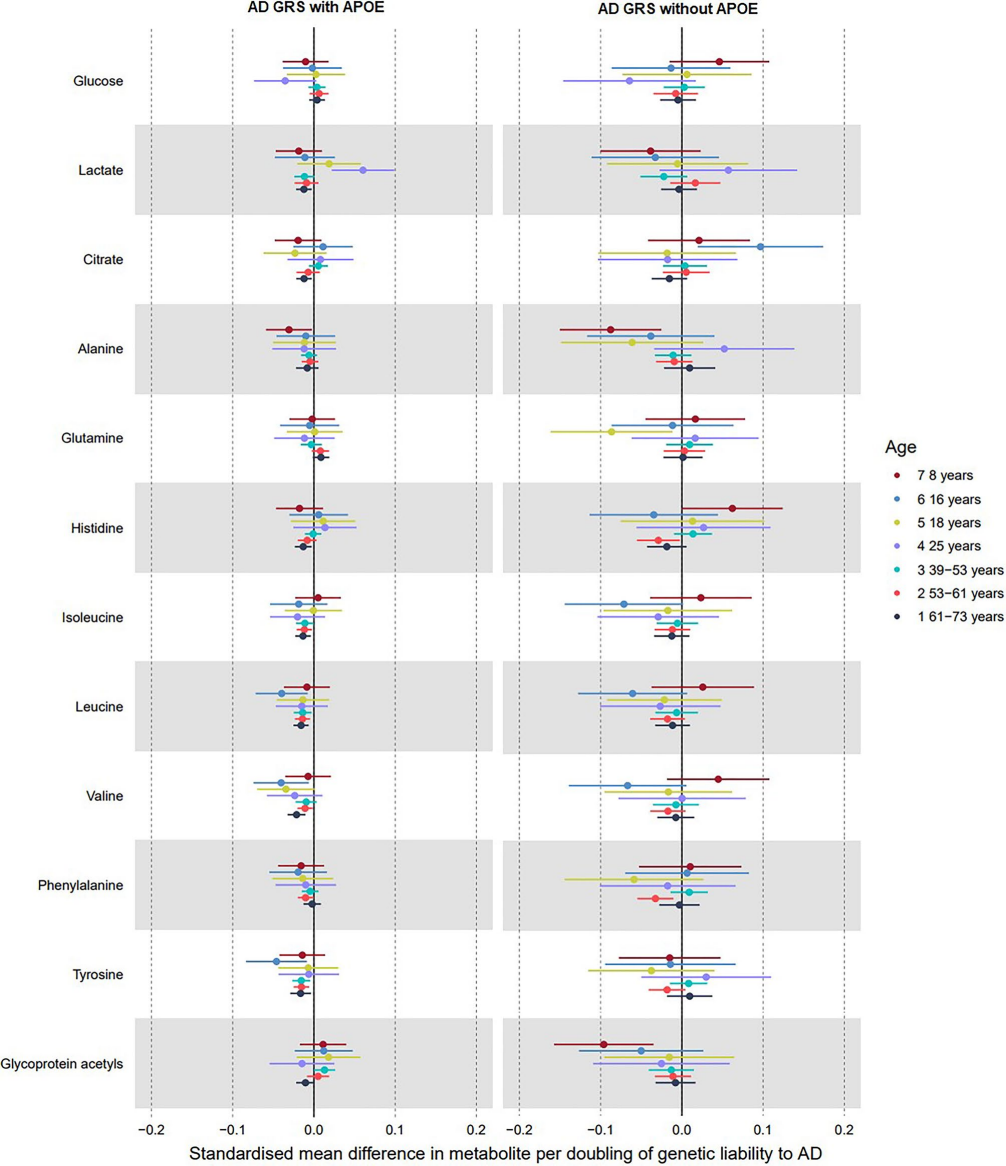
Forest plot showing the estimated effect of higher AD liability on main non-lipid metabolites (left panel including *APOE* variants; right panel excluding them). UK Biobank MR estimates are from the inverse variance weighted model.

Supplementary Tables 6 and 7 show associations of the AD GRS with metabolites in ALSPAC. Supplementary Tables 8–11 show effects of genetic liability to AD on the metabolites in the UK Biobank for the IVW, MR-Egger, weighted median and weighted most models, respectively. Overall, when strong evidence was observed for effects of the AD GRS on metabolites (i.e., confidence intervals did not span the null), the direction and magnitude of the effect sizes remained consistent across the life course (Figs. 2, 3 and 4). One exception to this was for the main lipid metabolites in UK Biobank, where there was generally attenuation of effect sizes towards the null in the older age tertile. UK Biobank estimates were largely consistent across MR sensitivity models, with expectedly wider confidence intervals for MR Egger estimates and narrower confidence intervals for weighted median and weighted mode estimates compared to IVW. In addition, there was little evidence to suggest the AD GRS was associated with BMI, height, smoking, alcohol consumption, physical activity, maternal and paternal educational attainment, or maternal or paternal occupational social class in ALSPAC (Supplementary Table 5). For all metabolite subcategories, there was substantial attenuation of beta values towards the null, with some loss of statistical power when excluding *APOE* variants from the GRS. This suggests that results were largely driven by *APOE* variants.

### Genetic liability to AD and lipid traits

Associations between genetic liability to AD and lipid metabolites are illustrated in Fig. 2 and Supplementary Tables 6–11. Of all metabolite subtypes, when including *APOE* variants in the GRS, lipid traits demonstrated the most consistent and largest magnitude of association with higher AD liability (Fig. 2). There was evidence of positive associations between higher AD liability and the following lipid metabolites across the life course: serum total cholesterol, very-low density lipoprotein (VLDL) cholesterol, remnant cholesterol, low-density lipoprotein (LDL) cholesterol, esterified cholesterol, free cholesterol, apolipoprotein B, ratio of apolipoprotein B to apolipoprotein A1, and sphingomyelins. For these same lipid metabolites, UK Biobank effect estimates from IVW models remained positive but attenuated towards the null across higher age tertiles, though confidence intervals overlapped (e.g., LDL cholesterol, youngest: 0.15 SD; 95% CI 0.07, 0.23, intermediate: 0.13 SD; 95% CI 0.07, 0.20, oldest: 0.10 SD; 95% CI 0.05, 0.16). Estimates from weighted mode and weighted median models in UK Biobank showed similar trends across age tertiles for total, VLDL and LDL cholesterol and apolipoprotein B, but with non-overlapping confidence intervals between the intermediate and oldest tertile. Across both cohorts, there was evidence of an inverse effect of AD liability on high-density lipoprotein (HDL) cholesterol, that was closer to the null at higher ages. There was no association with triglycerides in HDL at any ALSPAC time point. However, the effect estimates from IVW models for AD liability in UK Biobank were negative for triglycerides in HDL and increased in magnitude with age, which was consistent across sensitivity models. AD liability had no effect on apolipoprotein A1 in ALSPAC but was there was evidence of an inverse effect in UK Biobank that did not differ across age groups. For sphingomyelins, there was consistent evidence of a positive effect of AD liability in both ALSPAC (e.g., 25 years: 0.07 SD; 95% CI 0.04, 0.09) and UK Biobank, without differences by age. There was evidence of a positive effect of AD liability on triglycerides in LDL at all ages, but with a more modest effect in the oldest tertile of UK Biobank.

### Genetic liability to AD and fatty acids

Associations between genetic liability to AD and fatty acid metabolites are illustrated in Fig. 3 and Supplementary Tables 6–11. At each of the seven time points, there was evidence to suggest that when including *APOE,* higher AD liability had a strong positive effect on many fatty acid (FA) metabolites. The largest magnitudes of associations were observed for total FA, linoleic acid, omega-3 FA, omega-6 FA, polyunsaturated FA, monounsaturated FA, and saturated FA (Fig. 3), and the ratio of linoleic acid to total FA. Estimated effects of AD liability on other corresponding FA ratios were attenuated towards the null. Overall, for FAs there was a trend of smaller effect sizes as age increased; for some (e.g. total FAs, monounsaturated and saturated FAs), the confidence intervals of the oldest age tertile did not overlap with the intermediate age tertile (e.g. IVW: total FAs, oldest tertile: 0.02 SD, 95% CI 0.01, 0.03, youngest tertile: 0.06 SD, 95% CI 0.04, 0.08). The effect of liability to AD on the ratio of docosahexaenoic acid (DHA) to total FAs turned from negative to null with increasing age across both cohorts, whilst the effect on the ratio of linoleic acid to total FAs remained consistent across all age groups.

### Genetic liability to AD and non-lipid traits

Associations between genetic liability to AD and non-lipid metabolites are illustrated in Fig. 4 and Supplementary Tables 6–11.

#### Glycolysis-related traits

In ALSPAC, effect sizes for the association between higher AD liability and glycolysis-related traits (glucose, citrate, and lactate), both including and excluding *APOE* variants, centre around zero, and estimates were imprecise and generally close to the null. Considering associations including *APOE* variants, within the oldest UK Biobank tertile, there was evidence of an inverse effect of AD liability on citrate in all models. In ALSPAC, associations of AD liability with lactate were more positive at older ages (25 years: 0.04 SD; 95% CI 0.01, 0.06) with largely overlapping confidence intervals for each age group, but effect estimates in all UK Biobank age tertiles were negative (e.g., IVW, oldest: − 0.01 SD; 95% CI − 0.02, 0.00).

#### Amino acids and inflammation

Of all metabolite subcategories, amino acids (including the branched chain amino acids (BCAAs) isoleucine, leucine, and valine) demonstrated the weakest associations with higher AD liability including *APOE* variants. In ALSPAC, there were no consistent positive associations with any amino acids at any time point. In UK Biobank, higher liability to AD including *APOE* variants had an inverse association with some amino acids—either in all three age groups (e.g., tyrosine, leucine and isoleucine) or with the strongest evidence in the oldest group alone (histidine, valine, GlycA). There was also no association of higher AD liability with glycoprotein acetyls, a marker of inflammation, at any time point in ALSPAC or UK Biobank IVW models.

### Additional analysis: comparing metabolic profiles of ε4 and ε2 carriers with ε3 homozygotes

Given our primary results indicate that most associations are largely driven by *APOE* variants, we evaluated the metabolic profiles of participants who were ε2 and ε4 carriers (one or two copies) compared with ε3 homozygotes, in ALSPAC and UK Biobank.

#### Lipids

**Figure 5:**
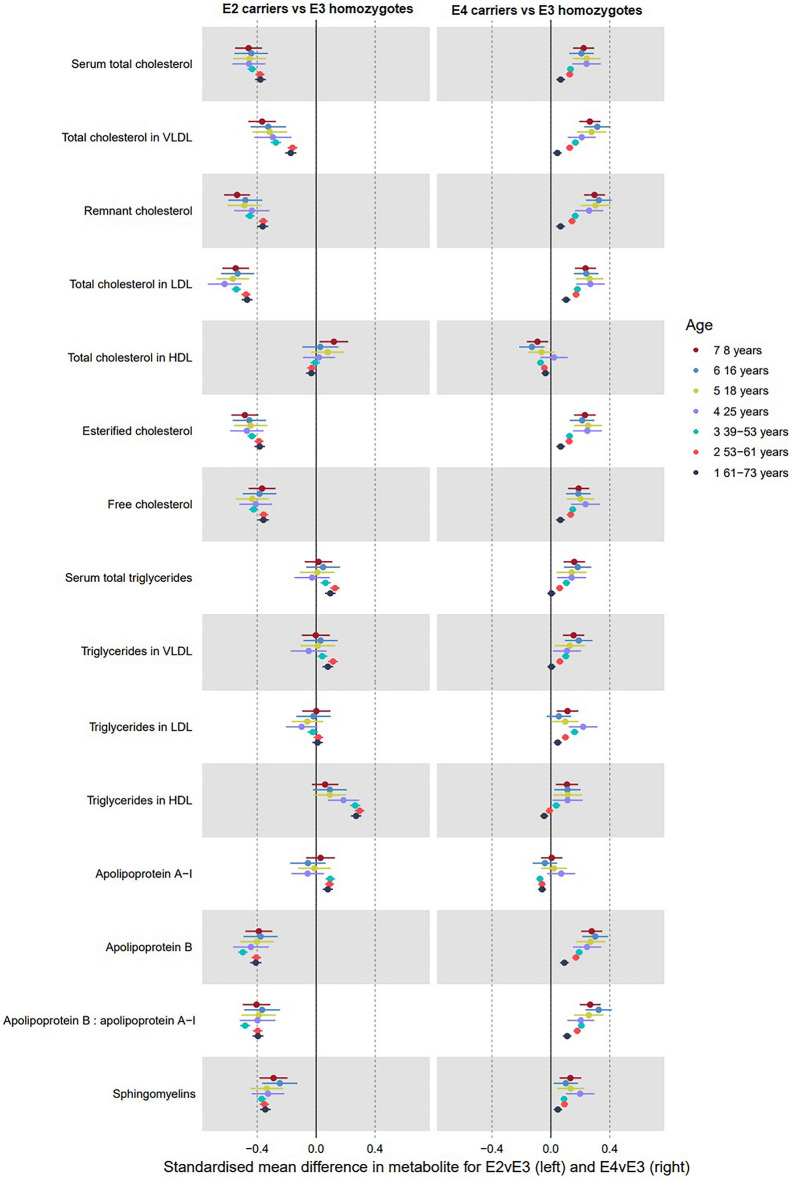
Forest plot showing the estimated effects of ε4 carriage (left panel; reference ε3 homozygotes) and ε2 carriage (right panel; reference ε3 homozygotes) on lipid metabolites.

Figure 5 and Supplementary Table 12 shows effects of APOE on lipid metabolites. There was evidence that ε4 carriers had, on average, higher levels of all lipids than ε3 homozygotes at all time points, with the exception of HDL cholesterol and Apolipoprotein A-I. The magnitude of effects of *APOE4* ε4 carriage on lipid metabolites generally decreased at higher ages (noting that differences in total triglycerides, triglycerides in VLDL, and triglycerides in HDL were very close to null in the oldest age tertile of the UK Biobank). ε4 carriage was associated with lower total HDL cholesterol and Apolipoprotein A-I, but the magnitude of effect was smaller than for other lipids and confidence intervals crossed the null for the two older timepoints in ALSPAC for HDL, and for all ALSPAC timepoints for Apolipoprotein A-I. ε2 carriage (relative to ε3 homozygosity) was associated with lower levels of total cholesterol, VLDL, remnant cholesterol, LDL, esterified cholesterol, free cholesterol, Apolipoprotein B, Apolipoprotein B to Apolipoprotein A-I ratio, and sphingomyelins. Magnitudes of effects were generally consistent across all timepoints. ε2 was also associated with higher HDL, but confidence intervals crossed the null before mean age 25. There was little evidence of an effect of ε2 on other lipid metabolites.

#### Fatty acids

**Figure 6:**
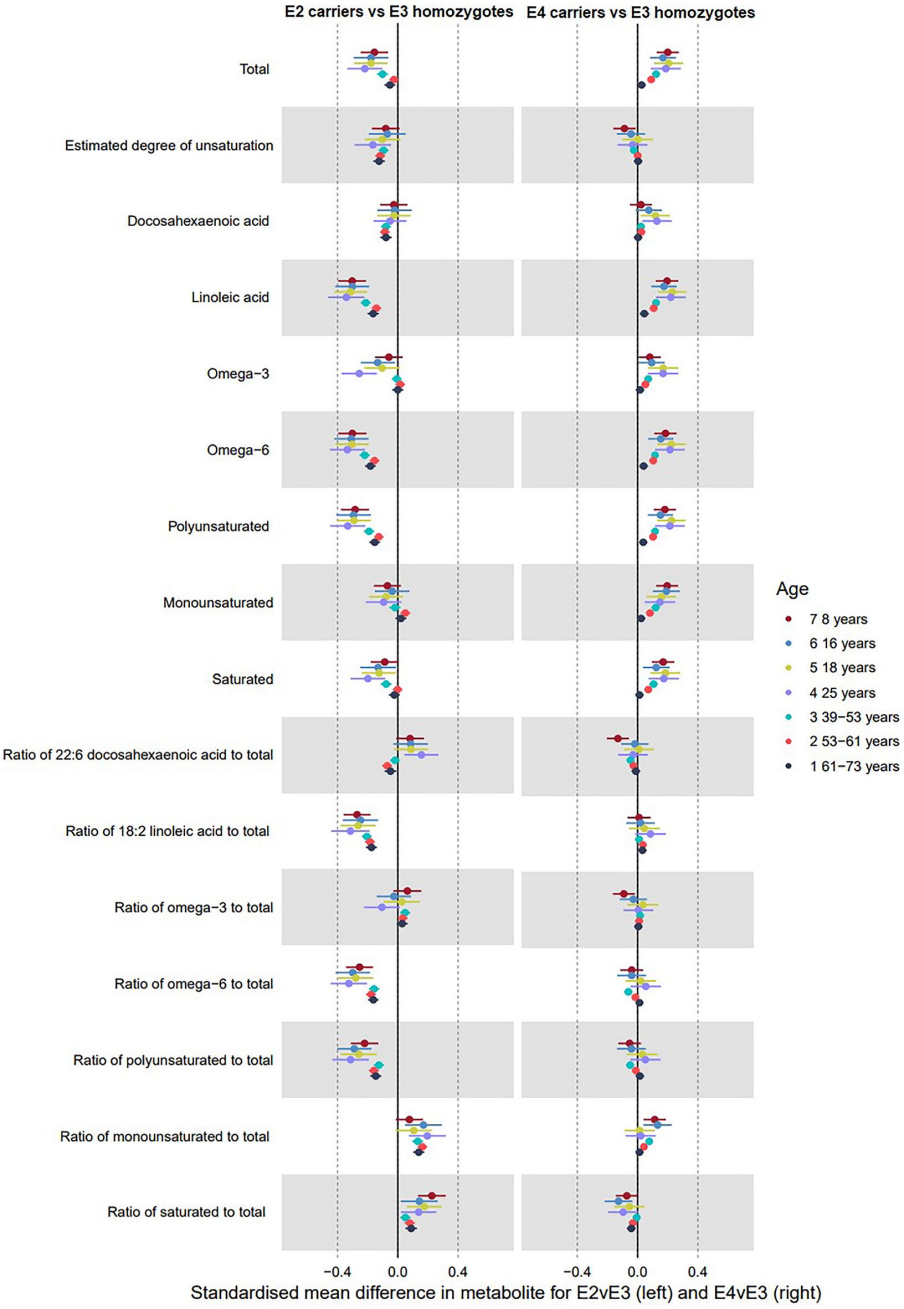
Forest plot showing the estimated effects of ε4 carriage (left panel; reference ε3 homozygotes) and ε2 carriage (right panel; reference ε3 homozygotes) on fatty acid metabolites.

Figure 6 and Supplementary table 13 shows effects of APOE on fatty acid metabolites. ε4 carriage was associated with higher levels of total FAs, linoleic acid, omega-3 and 6, poly- and mono-unsaturated FAs, saturated fatty acids, and the ratio of saturated to total FAs. ε2 carriage was associated with lower levels of total FAs, linoleic acid, omega-6, polyunsaturated, saturated, and the ratios of linoleic, omega-6 and polyunsaturated to total FAs. For ε4 carriage, the magnitudes of effects for each metabolite were generally higher during childhood and early adulthood, and attenuated with each increasing age tertile in UK Biobank. For ε2 carriage, magnitudes of effect were generally larger in childhood and early adulthood, and attenuated in older adulthood (UK Biobank) but with similar magnitudes in each of the age tertiles.

#### Non-lipids

**Figure 7:**
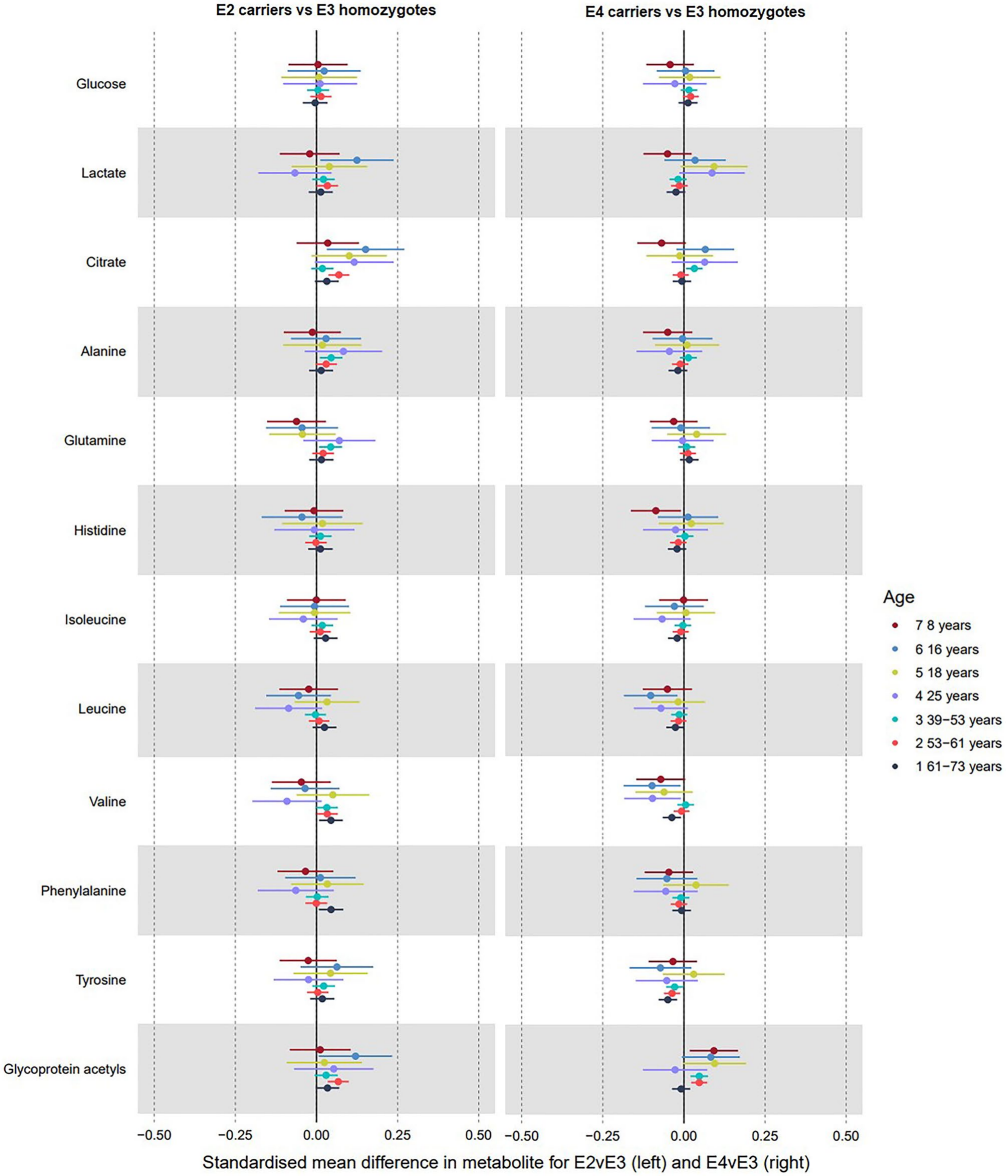
Forest plot showing the estimated effects of ε4 carriage (left panel; reference ε3 homozygotes) and ε2 carriage (right panel; reference ε3 homozygotes) on non-lipid metabolites.

Figure 7 and Supplementary Table 14 shows effects of APOE on non-lipid metabolites. There was very little consistent evidence that ε4 or ε2 affected non-lipid metabolites with most estimates varying around the null. Tyrosine was lower with ε4 carriage in UK Biobank. In the oldest tertile of UK Biobank only, ε4 and ε2 carriage appeared to have opposing effects on circulating valine. There was also some evidence to suggest ε4 carriage was associated with higher glycoprotein acetyls until ages 39–53 years, but this attenuated to the null in the middle and oldest age tertiles of UK Biobank.

## Discussion

This study estimated the effects of genetic liability to AD on the circulating metabolome measured across early life and into adulthood, revealing potential early stages of AD pathophysiology. Our most striking finding is the pronounced and enduring influence of the ε4 and ε2 isoforms on pro- and anti-atherogenic lipid and fatty acid traits, respectively, which was evident from childhood and persistent into later adulthood. Excluding *APOE* variants, the remaining AD genetic liability modelled here had little impact on the circulating metabolome across life. Many effects of ε4 and ε2 carriage on metabolites appeared to attenuate in older age groups (particularly in those aged 61–73 years). There was also very little evidence to suggest AD liability affects glycolysis- and inflammatory-related traits, suggesting that AD liability is more specifically reflected in lipid metabolism due to the impact of ApoE on a wide range of lipid fractions.

It has been hypothesised that higher genetic liability to AD (including *APOE* ε4 variants) may impact AD risk via its effect on atherosclerosis. This is supported by both comparable enrichment of plasma lipid subtypes for AD and CVD^31^ and demonstration here of the strongest positive associations being for the proatherogenic traits LDL cholesterol, apolipoprotein B and ratio of apolipoprotein B to apolipoprotein A1. It has also been shown that elevated LDL cholesterol is associated with increased cerebral amyloid deposition^32^. We found evidence that associations of HDL cholesterol and its major constituent apolipoprotein A1 with higher AD liability were weakly negative. These results complement the findings of an MR study suggesting a protective effect of HDL cholesterol and apolipoprotein A1 with respect to AD risk^33^. That said, other MR studies have found little evidence of an effect of lipid-related traits on AD risk^34^.

We found evidence of a positive association between higher AD liability and sphingomyelins levels that was consistent across all age groups, and appears to be specific to carriage of AD risk variants in *APOE*. Sphingolipids are a class of lipids, of which sphingomyelins are members^35^. They are often found in neuronal myelin & may be linked to neurodegeneration; a study of post-mortem brains, CSF and plasma implicated sphingomyelin perturbations in AD pathophysiology^36^, and a targeted metabolomics study of blood and brain found that increased sphingomyelin levels correlated with AD severity, tracking disease progression from prodromal to preclinical stages^37^. Their use as a potential early AD biomarker should be further explored.

We show that the effect of higher AD liability on triglyceride levels in VLDL, HDL and total triglycerides weakens with age. This could reflect increased lipid-lowering medication use with age (e.g., statins), which would be expected to be highest among ε4 carriers due to higher dyslipidaemia incurred by the variant. It could also reflect, at least in part, survival bias, whereby individuals with higher dyslipidaemia and associated sequalae are at increased risk of premature mortality. Disease pathogenesis leading to dietary changes (an established part of the AD prodrome) may also explain some of the changes to metabolites heavily influenced by dietary intake at later ages, and this is perhaps most relevant for fatty acids and proteins. The magnitude of effect is considerably less than what was observed for a recent untargeted lipid profiling study by Bernath et al. which concluded, as we did here, that AD-mediated effects on triglycerides were specific to carriers of *APOE* ε4^38^, apart from triglycerides in HDL which were, on average, higher for ε2 carriers.

When including *APOE* variants, strong positive associations were observed between AD genetic liability and total FAs, linoleic acid, omega-6 FAs and polyunsaturated FAs. Corresponding FA ratios, which may better reflect FA biology^22^, demonstrated an attenuated, yet still positive associations with higher AD liability. Aside from functioning as membrane constituents and energy sources, FAs mediate inflammation^39^, a process central to the pathogenesis of both CVD and AD^40^. Linoleic acid has previously been associated with the extent of AD neuropathology in a nontargeted metabolomics study^41^, though small sample size and confounding limit causal inference.

Except for fatty acid traits, there was very little evidence that higher AD liability affects non-lipid (e.g. glycolysis and amino acid) metabolites in our study. Type 2 diabetes, defined as elevated plasma glucose, is hypothesised to be a risk factor for AD, although MR studies to date have not supported a causal association^42,43^. Diabetes mechanisms may mediate the pathological effects of the ε4 genotype^44^ and influence cerebral glucose metabolism^45^. Results from a prospective cohort study with several decades of follow-up suggested that plasma glucose dysregulation is only evident in ε4 carriers from midlife onwards^46^. Despite this, even in the oldest UK Biobank tertile, we observed little evidence of effect of AD liability on glucose. The effect of AD liability on lactate was positive at older ages in ALSPAC, but inverse or null in UK Biobank age tertiles. Increased lactate in the CSF and brain has been associated with higher AD risk, the degree of perturbation correlating with extent of neurodegeneration^47^. In vitro evidence suggests that this trend may be ε4-mediated^48^. The lack of consistency of effect of AD liability on lactate levels across different life-stages suggests limits to its clinical utility as a biomarker of early disease. Evidence for the role of BCAAs in AD is inconclusive. Observationally, increased BCAA levels appear to protect against AD^49^, supported by our inverse effect estimates for AD liability on BCAAs observed in UK Biobank. Increased BCAAs have, however, been robustly associated with increased diabetes risk in MR analysis^50^. The absence of association between increased BCAAs and higher AD liability in this study perhaps suggests that the link between BCAAs and AD is mechanistically distinct from pathways of glucose and insulin metabolism. This, however, contradicts results from a recent MR analysis that concluded those predisposed to raised plasma isoleucine levels are at an increased rather than decreased risk of AD^51^.

### Strengths and limitations

Prior studies have been limited in their ability to determine whether metabolic perturbations were a cause or consequence of disease activity. However, given the use of genetic instruments for AD, and the young age of ALSPAC participants, observed effects in this cohort are likely to precede clinical AD and are therefore not consequences of AD pathophysiology. Moreover, although it is known that *APOE* variants are associated with differences in lipid metabolism, this is the first study to compare the magnitude of these effects to other non-lipid metabolites, elucidate the protective effects of ε2 carriage in greater detail, and conduct temporal profiling of these perturbations across the life course to identify the earliest ages at which they can be observed.

Our analyses are underpinned by three core instrumental variable (IV) assumptions that must be satisfied for results to be valid. The first assumption of robust association between the IV and trait of interest was fulfilled given the large GWAS sample size and inclusion of SNPs relating to genes with known a priori biological function in relation to AD (*APOE*). The second assumption is that of no confounders of the IV and the outcome. This was addressed to the extent possible here by using a largely ancestrally homogenous population (> 96% white ethnicity). The final assumption is that there is no association of genetic instruments with the outcome, except via the exposure of interest. Our UK Biobank results largely demonstrated consistency across pleiotropy-robust models, indicating that horizontal pleiotropy is unlikely to be causing bias in our observed effects. There are several limitations to our study. The lack of ancestral diversity in ALSPAC (96% white) and UK Biobank (only Europeans analysed to avoid genetic confounding) limits the generalizability of results to diverse populations, though reduces the potential for confounding by population stratification. Given that ε4carriage affects AD risk more in those of European ancestry than those of African American or Hispanic ancestry^52^, future studies could investigate the extent to which *ε4* carrier status influences the metabolome for other populations to help understand the reasons for ε4-AD risk differences across ancestral groups. Despite the central-peripheral flux of metabolites via the blood–brain barrier, previous studies have noted that the AD molecular profiles of plasma and CSF are divergent^53^. Therefore, the extent to which inferences regarding central AD pathophysiology can be made from this study should be considered. Future work should compare the effect of higher AD liability on plasma and CSF metabolites, although such data do not yet exist at scale. UK Biobank and the ALSPAC 8-year metabolite measurements were taken from non-fasted blood samples, whilst samples from all other timepoints were fasted, which potentially limits the comparability of UK Biobank and age 8 with the other ages. Another limitation is the targeted nature of the Nightingale metabolomics platform, which focuses on metabolites previously identified to be of clinical interest, most of which are lipids. An untargeted approach would allow for discovery of unknown biomarkers, including those beyond the lipid classes, of AD liability. The potential for selection bias is a plausible limitation to our study. However, for both ALSPAC and the UK Biobank, AD liability has been shown to be associated with non-participation^54,55^. Thus, selection bias would be anticipated to cause bias towards the null. Lastly, vertical pleiotropy could plausibly explain some of our findings. This is where perturbations in one metabolite causally influences another metabolite, but AD liability is not causally associated with the latter (the latter only changes as a result of changes in the former, which is the causal biomarker).

## Conclusions

The results of this study support pronounced, and in many cases age-varying, effects of *APOE* ε4 and ε2 in producing early metabolic signatures of higher AD liability, many decades before the typical clinical presentation of late onset AD. Such metabolic characterisation of AD risk requires further examination within different cohorts and other study designs to strengthen evidence and improve understanding of AD pathophysiology, with implications for the prediction and prevention of the disease.

### Supplementary Information

Supplementary Tables.

Supplementary Figure 1.

## Data Availability

Individual-level ALSPAC data are available following application. This process of managed access is detailed at www.bristol.ac.uk/alspac/researchers/access. Summary-level GWAS results can be accessed through the IEU-OpenGWAS platform, accessible at https://gwas.mrcieu.ac.uk/datasets/?gwas_id__icontains=met-d. Summary statistics for the Kunkle et al. meta-analysis are available at: https://www.niagads.org/datasets/ng00075.

## Abbreviations

AD: Alzheimer’s disease
*APOE*: Apolipoprotein E
CSF: Cerebral Spinal Fluid
GWAS: Genome-wide association studies
SNP: Single nucleotide polymorphism
ALSPAC: Avon longitudinal study of parents and children
GRS: Genetic risk score
^1^H-NMR: Proton nuclear magnetic resonance
MR: Mendelian randomization
IVW: Inverse variance weighted
FA: Fatty acid
HDL: High-density lipoprotein
LDL: Low-density lipoprotein
VLDL: Very low-density lipoprotein
BCAA: Branched chain amino acids
IV: Instrumental variable
CVD: Cardiovascular disease
