# Supplementary material for: Life course plasma metabolomic signatures of genetic liability to Alzheimer’s disease
Source: Sci Rep. 2024 Feb 16;14:3896. doi: 10.1038/s41598-024-54569-w (PMC10873397; doi:10.1038/s41598-024-54569-w)
Supplement: Supplementary file 2 — Supplementary Figure 1. [file 41598_2024_54569_MOESM2_ESM.docx]

**Online Supplement**

**Details of *ALSPAC***

Eligibility of pregnant women for inclusion to ALSPAC, population-based multi-generational birth cohort study, was based on residence in a defined area of South West England and an estimated delivery date between 1^st^ April 1991 and 31^st^ December 1992^1^. Recruitment occurred in four phases yielding a total of 15,454 pregnancies and 15,589 foetuses, 14,901 of whom were alive at one year^2,3^. Ethical approval for the study was obtained from the ALSPAC Ethics and Law Committee and the Local Research Ethics Committees. Consent for biological samples has been collected in accordance with the Human Tissue Act (2004). The study website contains details of all available data through a searchable data dictionary and variable search tool (<http://www.bristol.ac.uk/alspac/researchers/our-data/>). Study data were collected and managed using REDCap electronic data capture tools hosted at the University of Bristol^24^. REDCap (Research Electronic Data Capture) is a secure, web-based software platform designed to support data capture for research studies.

***Supplementary Tables 1-9 are in an excel file***

***Supplementary Figure 1***


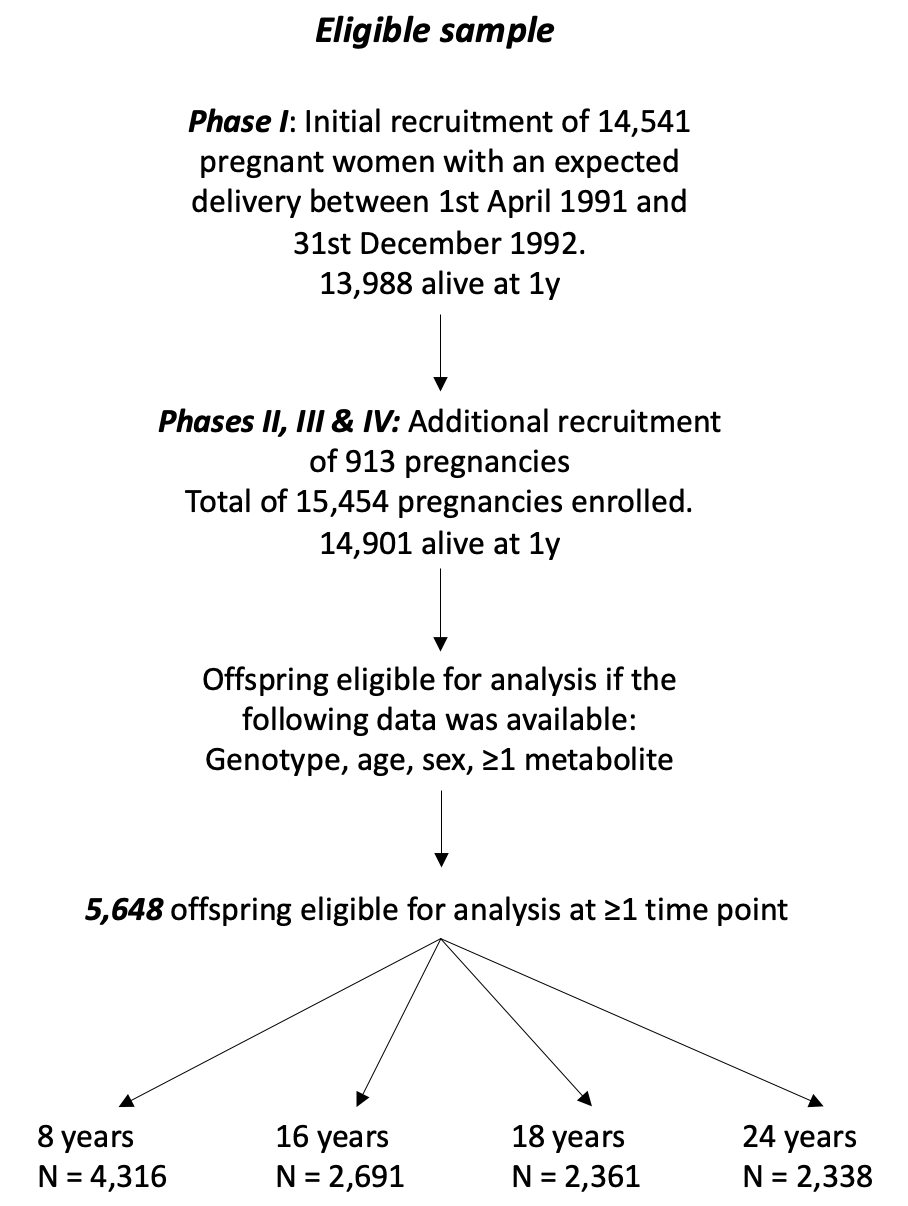


***Supplementary Figure 1*** Flow chart to demonstrate eligibility criteria for inclusion to ALSPAC analyses

**References**

1. Fraser A, Macdonald-Wallis C, Tilling K, Boyd A, Golding J, Davey Smith G, et al. Cohort Profile: The Avon Longitudinal Study of Parents and Children: ALSPAC mothers cohort. Int J Epidemiol. 2013 Feb;42(1):97–110.

2. Boyd A, Golding J, Macleod J, Lawlor DA, Fraser A, Henderson J, et al. Cohort Profile: The ‘Children of the 90s’—the index offspring of the Avon Longitudinal Study of Parents and Children. Int J Epidemiol. 2013 Feb;42(1):111–27.

3. Northstone K, Lewcock M, Groom A, Boyd A, Macleod J, Timpson N, et al. The Avon Longitudinal Study of Parents and Children (ALSPAC): an update on the enrolled sample of index children in 2019. Wellcome Open Res. 2019 Mar 14;4:51.
